# A Kinetic Ecological Approach to Beauty Perception: A Perspective Review on the Case of Symmetry and the Golden Ratio

**DOI:** 10.1111/ejn.70119

**Published:** 2025-04-25

**Authors:** Marco Iosa, Maria Pia Lucia, Claudia Salera

**Affiliations:** ^1^ Department of Psychology Sapienza University of Rome Rome Italy; ^2^ SmArt Lab IRCCS Santa Lucia Foundation Rome Italy

**Keywords:** aesthetics, affordance, art, perception, psychology, vision

## Abstract

In the 1970s, the psychologist J.J. Gibson developed an “ecological approach to visual perception”, suggesting that humans perceive the environment exploiting environmental affordances—surrounding invariant features that define possible individuals–object interactions—without top–down mediation of cognitive processes. Shepard extended this approach, suggesting that common environmental features are internalized defining perceptual constraints, such as the circadian rhythm, three‐dimensional space, and gravity. In this perspective review, we apply these concepts to neuroaesthetics and beauty perception, discussing how the internalization of invariants may influence our perception of beauty. Within this framework, special emphasis was placed on symmetry and golden ratio, typically considered as two benchmarks for beauty, and two geometrical features that can be considered as environmental affordances. Moreover, we suggest that kinetic aspects play a role in beauty perception of these proportions, particularly by the internal model of gravity.

## Graphical abstract

- Perception through Environmental Affordances: Objects offer invariant features for interaction, symmetry, and golden ratio could be aesthetic affordances.
- Common Environmental Features (circadian rhythm, 3D environment, gravity etc.) could be internalized, leading individuals to expect regularities in aesthetic perception.
- Internalization of physical laws or environmental features (i.e., symmetry, golden ratio) are here hypothesized as beauty drivers that may influence our aesthetic preferences due to their kinetic aspects affording implicit interactions.

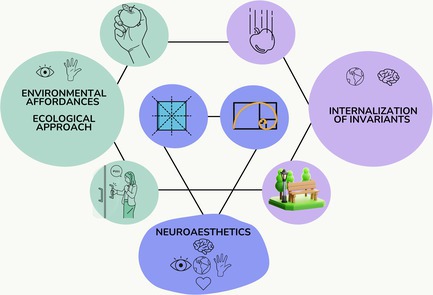

**Abbreviations**
GR: golden ratio
SPN: sustained posterior negativity

## 1 Introduction

### 1.1 Is Beauty in the Eye of the Beholder?

“Beauty is in the Eye of the Beholder” is a well‐known quote originally penned by Margaret Wolfe Argles Hungerford in her novel *Molly Bawn* (Hungerford 1878), which tells the story of a flirtatious Irish girl who arouses her lover's jealousy and naively ignores social conventions.

Numerous researchers have investigated the scientific validity of this insightful phrase. Is beauty a characteristic of an object, a person, an animal, a place, and so being objective or is it “in the eye of the beholder” and hence subjective? Chen et al. (1997) defined this dualism as “the owner vs. observer hypothesis”. Some researchers support the idea that beauty is an objective quality. For example, studies have found that beautiful faces consistently exhibit characteristics such as symmetry (Scheib et al. 1999), average generic features (Borelli and Berneburg 2010), above‐average sexually dimorphic characteristics (Barber 1995), and a youthful aspect (Kwart et al. 2012). Conversely, other studies report significant variability in aesthetic preferences among individuals, depending on personality (Fisak et al. 2007), behaviors (Forestell et al. 2004), and cultural standards (Kedia et al. 2014), suggesting that beauty is subjective and that individual differences must be taken into account (Friedenberg 2019).

A functional magnetic resonance imaging study observed participants observing pictures of famous artworks (sculptures). The results indicated that different brain structures could be associated with objective and subjective beauty. Specifically, in naïve observers, perception of beauty is mediated by two non‐mutually exclusive processes: a joint activation of separate sets of cortical neurons—triggered by intrinsic parameters of the stimuli—and the insula would activate for objective beauty, whereas subjective beauty would rely on the amygdala, driven by one's own emotional experience. Both processes are not mutually exclusive and coexist to elicit our sense of beauty (Di Dio et al. 2007).

Counting for neural correlates of beauty, Ramachandran and Hirstein (1999) proposed a structured framework for aesthetic perception outlining eight laws of artistic experience, which describe how certain visual and perceptual mechanisms are optimized to elicit aesthetic pleasure. These principles suggest that our neural architecture is predisposed to respond to specific artistic features, making our art appreciation partly hardwired in the brain. Their framework established a foundation for systematic inquiry into neuroaesthetics by linking perceptual processing with evolutionary function.

But although Ramachandran and Hirstein's approach laid a crucial foundation for understanding neural aspects of aesthetic perception, it also limitedly conceptualized art appreciation as passive visual processing, focusing on how the brain detects and enhances stimulus‐driven properties (e.g., contrast, symmetry, perceptual grouping) to maximize aesthetic pleasure. However, aesthetic is not solely a function of passive visual perception; it also emerges through dynamic interaction with the environment, engaging sensorimotor systems, spatial cognition, and embodied interactions (Gibson 1979; Freedberg and Gallese 2007).

The kinetic ecological approach proposed here extends this understanding by emphasizing the role of affordances, internalized invariants, perceived kinetics, and embodied interaction in shaping aesthetic appreciation. Rather than considering visual aesthetics as a set of static properties, this perspective highlights how sensorimotor engagement, spatial dynamics, and motion‐based perception underlie the aesthetic experience. In this view, the act of looking is not merely a receptive process but an active exploration. This synthesis moves beyond passive observation to incorporate kinetic laws and environmental affordances as essential components of aesthetic engagement. Exposure to environmental physical invariants (such as gravity) can be the evolutionary rationale at the basis of the proposed kinetic approach to neuroaesthetics. On the other hand, the internalization of these invariants has been already demonstrated in neurophysiological studies of visual perception (Zago et al. 2004; De Bartolo et al. 2022).

### 1.2 Is Beauty an Unaware Perception?

Another interesting question about beauty is whether aesthetic attractiveness is processed with or without conscious awareness. Research suggests that beauty can be processed with brief exposure to faces (13 ms with highly attractive faces). Such a fast appraisal may indicate that attractiveness is evaluated in absence of conscious awareness (Olson and Marshuetz 2005; Hung et al. 2016). Moreover, people (especially males) tend to spend more time looking at beautiful faces, suggesting that attractive faces attract attention more effectively (Aharon et al. 2001). Other studies showed that unattractive faces capture greater attention than moderately attractive faces, but beautiful faces elicited greater hand movements compared to both unattractive and moderately attractive faces (Valuch et al. 2015). However, other researchers argued that the appraisal of face attractiveness requires conscious awareness (Tsikandilakis et al. 2019).

Research on unconscious beauty processing has not only focused on faces, but has also extended to broader visual features, such as symmetry, proportion, and compositional balance. Certain aesthetic principles, particularly symmetry, appear to be preferred spontaneously, without requiring deliberate thought. Psychological and neuroscientific studies suggest that humans exhibit an implicit preference for symmetrical patterns in nature, abstract designs, and artistic compositions, likely because symmetry facilitates perceptual fluency and efficient processing (Reber et al. 2004; Reber and Schwarz 2006; Makin et al. 2012; Bertamini et al. 2013).

The scientific exploration of unconscious aesthetic preferences can be traced back to Gustav Fechner (1876), a pioneer in experimental aesthetics, who sought to determine whether certain compositional structures, such as the golden ratio (GR), hold an intrinsic aesthetic appeal. In his studies, he asked participants to create or select aesthetically pleasing shapes and compositions, finding mixed results. Some studies did report a preference for the GR, others failed to provide conclusive evidence (Höge 1995, 1997). The idea that aesthetic preferences are biologically ingrained has been further challenged by McManus and Weatherby (1997). Their research argues that preferences for compositional proportions (including the golden section) are not necessarily innate but are shaped by cultural exposure, learned associations, and familiarity. This perspective aligns with cognitive and cultural theories of aesthetic perception, which propose that beauty is influenced by exposure, learning, and social context. While certain low‐level visual properties (e.g., symmetry, color harmony) may elicit an immediate, unconscious response, more complex aesthetic judgments, such as preferences for specific artistic styles or proportions, may likely involve higher‐order cognitive processes and cultural conditioning.

### 1.3 Is Beauty an Emotion?

The perception of beauty is not merely a cognitive or perceptual process; it is also deeply intertwined with emotion and affective experience. Throughout history, philosophers and intellectuals have debated the origins of aesthetic pleasure. One of the most influential figures in this sense is the philosopher Immanuel Kant (1790), who proposed that judgments for beauty are simultaneously sensorial, emotional, and intellectual. In his book *Kritik der Urteilskraft* (i.e., *Critique of Judgement*), he argued that the experience of beauty is a feeling that an object resonates with our mental structures, but only at an abstract level that does not involve recognition through a specific concept. In other words, Kant considered the experience of beauty as emerging from an intrinsic harmony between perception and cognition.

Starting from his assumption, contemporary cognitive theories have further proposed to consider beauty as a pleasant emotion, which involves the detection of inherently pleasant processing dynamics, appraised as congruent with an active epistemic goal. For instance, Armstrong and Detweiler‐Bedell (2008) proposed that beauty functions as a distinct, pleasurable emotion, emerging when individuals perceive harmonious and fluently processed patterns that align with an active epistemic goal. According to this framework, the brain would effortlessly organize and interpret visual information, favoring symmetry, balance, and order due to its inherent processing tendencies. This perspective challenges the notion that aesthetic pleasure is merely a passive sensory response, instead it frames it as an active emotional experience deeply intertwined with cognitive learning and pattern recognition mechanisms.

Nevertheless, while beauty is often associated with positive emotions, research has also documented cases of negative affective experience in response to beautiful pieces of art. One prominent example is the Stendhal Syndrome, a psychosomatic condition involving rapid heartbeat, confusion, dizziness, and hallucinations that can emerge when an individual is exposed to artworks of exceptional beauty. This syndrome, named after the 19th‐century writer Stendhal, was first documented when he reported experiencing overwhelming emotions while visiting the Basilica of Santa Croce in Florence. Stendhal described a state of dizziness, extreme awe, and disorientation, which he attributed to the sheer beauty and historical depth of the artwork he was viewing. Although rare, this syndrome indicates that the emotional impact of beauty—even in the aesthetic art context—can lead to distress and negative effects, rather than pleasure (Arias 2019). This paradoxical nature of beauty (i.e., its ability to elicit both intense pleasure and overwhelming distress) has been explored in neuroscientific studies as well. Brain responses and emotional reactions to praised art have also been shown to elicit the activation of specific brain regions associated with strong emotional responses (e.g., amygdala, orbitofrontal cortex), as well as engagement of reward system (e.g., ventral striatum) (Lacey et al. 2011; see for a review Chatterjee and Vartanian 2014).

### 1.4 Beauty and the Best

People often show a preference for beautiful things and attractive faces, indicating a beauty‐related perceptual bias. This preference may generate a secondary bias, which leads to perceiving something beautiful as also good (Zhang et al. 2018). Indeed, evidence shows that attractive people usually benefit from enhanced positive perception (Griffin and Langlois 2006; Langlois et al. 2000) and income status with financial advantages (Judge et al. 2009). Singh et al. (2024) found a positive correlation between attractive females and perceived health and happiness, but not with increased age. Moreover, the beauty bias extends beyond positive stereotyping and can influence the accuracy of judgments, leading to considering as the best choice the most beautiful one. Lorenzo et al. (2010) found not only that attractive people are judged more positively, but also more accurately. This suggests that physical appearance may serve as a social cue that enhances interpersonal perception, possibly because attractive people elicit more attention and engagement; hence, leading observers to form more well‐rounded and precise impressions of them. However, while beauty can contribute to social and economic advantages, it can also generate bias with potentially harmful consequences. For instance, Mackelprang and Becker (2017) found that female sexual offenders were judged more leniently and received less punitive sentences compared to their male counterparts, a disparity that might be partially attributed to societal stereotypes associating female attractiveness with innocence or non‐threatening behavior. This evidence supports the idea that, while aesthetic appeal is a powerful social tool, its influence extends beyond surface‐level preferences, from hiring decisions and income inequality to legal judgments and interpersonal relationships. To understand these biases is crucial if we consider beauty as an ingrained preference, as it may contribute to highlight its pervasive role of appearance in human cognition and behavior, revealing both the benefits and the unintended consequences.

### 1.5 Aim of This Perspective Review

As discussed above, the concept of beauty raises numerous questions. This perspective review aims to contribute to the theoretical framework of beauty by addressing the controversies found in literature and suggesting a parallelism between beauty and the concept of affordance, as defined by Gibson (1979). Furthermore, according to the Shepard's (1984) theory about the internalization of the invariants, we propose how some invariant affordances may affect the perception of beauty while being linked to sensory motion system considering not only the static properties of a stimulus, but also its kinetic features. The theoretical framework proposed in this review is based on experimental findings reported in scientific literature from studies conducted in the fields of psychology, neuroaesthetics, neurophysiology, and visual perception. We begin by applying Gibson's (1979) theory of affordances to the perception of beauty, focusing on symmetry and the GR as affordances in visual perception. Then, we analyze how symmetry and the GR could function as internalized affordances according to the Shepard's (1984) theory of invariant internalization. Finally, we show how, from an evolutionary perspective, symmetry and the GR are environmental features linked to physical laws governing gravity and inertia, providing a possible kinetic explanation behind the aesthetic preference for symmetric and golden proportions.

## 2 The Affordance Hypothesis Extended to Beauty

Gibson defined the affordance of an object as “what the infant begins by noticing. The meaning is observed before the substance and surface, the color and form, are seen as such. An affordance is an invariant combination of variables …” (Gibson 1979). Consider a chair, for instance: it can be made of various materials such as wood, plastic, or metal, and may come in different colors and surfaces. However, the crucial aspect of a chair is its invariant functional property of affording sitting. A person perceives this functional value before noticing all the other variable features. Furthermore, affordances are relational, as they “cut across” the subject–object dichotomy: a chair only affords sitting because humans can use it for that purpose.

An affordance is neither an intrinsic property of the object, nor a subjective interpretation of its characteristics; rather, it is based on the possible interactions between the object and the subject. Gibson (1979) frequently compared affordances to the way an animal gathers information about its surroundings. For example, in the context of mate selection, animals detect affordances through their sensory modalities, including—touch, sound, smell, taste, and light. Moreover, Chemero (2003) expanded this view by suggesting that affordances may not be limited to sight, but can also be perceived through movement, emphasizing the role of dynamic touch. However, experimentally distinguishing between affordances and the information that specifies remains an ongoing challenge.

Another crucial aspect of affordances relies on locomotion. In this sense, Gibson (1979) emphasized that when we interact with the environment, our perception of the world is intrinsically linked to our sensorimotor abilities. For instance, to consider an animal perceiving a drinking bottle would imply the animal to be capable of interacting with the bottle in a meaningful way. This dynamic interplay evolved due to both environmental changes and evolution within us (Vara Sánchez 2022).

The concept of affordance, which to date holds significant untapped potential in empirical aesthetics, forms the foundation of the present study. This review focuses on the continuity between certain aesthetic experiences and everyday interactions, exploring how aesthetic visual cues can serve as meaningful vehicles in the social context.

Schwarz et al. (1991) extensively documented the impact of processing fluency (i.e., the ease with which information is processed) on our cognitive and affective responses. The authors demonstrated that fluently processed information enhances comprehension, strengthens memory retention, and significantly influences aesthetic preferences. Specifically, they found that when individuals process stimuli with more ease, they also tend to evaluate them more favorably.

Building on this foundation, Reber, Winkielman, and Schwarz (2004) explored how processing fluency affects our aesthetic judgments. They presented participants with a series of visual stimuli varying in complexity—some simple and familiar, others complex and unfamiliar. Participants rated these images based on beauty and likability, while processing ease was assessed through a pattern recognition task. Their findings revealed that stimuli that were easier to process were consistently rated as more beautiful and pleasing, highlighting the strong connection between processing fluency and aesthetic preference.

This insight into processing fluency aligns with the concept of aesthetic affordances, which refers to how objects or stimuli evoke positive emotional and cognitive responses based on their alignment with our perceptual expectations. While Reber et al. (2004) emphasized cognitive fluency in shaping beauty, Sánchez (2022) expanded this framework by integrating affective regulation into the understanding of aesthetic experiences.

In the study “What do aesthetic affordances afford?”, the author Sánchez built on the previous notion of “affective‐regulatory affordances” as coined by Krueger and Colombetti (2018), according to which affordances are not merely about how we interact with objects but also about how they influence our emotional states. In this sense, the author explains, affordances play a crucial role in regulating our affective experiences through dynamic interactions and actionable responses, such as seeking comfort from a friend when we are sad.

It has been suggested that aesthetic engagement can be interpreted as an “edge of action” where perceptual engagement occurs without immediate action (Brincker 2015). This state opens for a unique interaction with perceptual content that differs from our regular, practically engaged modes of perception. These “un‐actable” affordances would foster a deeper, difficult intimacy with perceptual experience and visual content. According to Brincker, certain artworks and natural elements offer unique affordances that foster a mode of engagement distinct from our typical environmental interactions. This “non‐interactive” perception encourages a deeper, more intimate experience with the perceptual content. Thus, the above‐mentioned Sanchez's work extends the discussion from processing fluency to include how stimuli that regulate emotions contribute to our overall aesthetic experience. This broader perspective highlights how both cognitive ease and emotional impact shape our perception of beauty, bridging the gap between cognitive psychology and aesthetic appreciation.

Sanchez's position on affective affordances is that not all the events we experience serve affective‐regulatory purposes, but that some of them can be particularly driven by that. The same applies to experiences that elicit aesthetic perception. As an attempt to bring aesthetics closer to affordances, the aesthetic perception can be understood from an embodied and situated perspective (Vara Sánchez 2022; Brincker 2015). Brincker identifies several dynamic elements that form an “aesthetic stance” and argued that artistic images, as well as certain elements from nature, present a mode of engagement that not only provides distinct affordances but also produces a sort of pause in our ongoing environmental interactions. This occurs because it enacts a non‐interactive mode of perception where the beholder perceives the beheld without reciprocal interaction. This notion extends to art as well; for example, in Gallagher's (2011) enactive‐embodied approach to motor theories of art engagement, certain artworks offer affordances that are neither actionable nor practical, nor do they invite direct interaction. These affordances contribute to the suspension of ordinary engagement with objects and help the observer to become more aware of their perceptual and cognitive possibilities (Gallagher 2011).

In recent years, it was put into discussion how aesthetic attractiveness may facilitate the way we visually process stimuli considered aesthetically pleasant. De Bartolo et al. (2022) investigated participants' detection of symmetry and the GR suggesting (for the first time) that these two geometrical proportions, often linked to beauty perception, can be considered as affordances. The authors hypothesized that symmetry and GR may function as visual affordances which are implicitly recognized and facilitate the processing of visual information. This aligns with the “processing fluency theory”, which states that increased fluency can contribute to the pleasantness of aesthetic experiences (Reber et al. 2004). Accordingly, in this work, we built on this hypothesis proposing further insights that consider these features as potential aesthetic affordances within visual perception.

Beyond the rapid, unconscious processing of facial beauty, another key factor influencing attractiveness is the direction of the light we assume is illuminating a face or object. Research on lighting direction and aesthetic judgment suggests that our perception of beauty is not only influenced by facial symmetry, contrast, and texture but also by the way light interacts with these features. This idea is detailed in Richard L. Gregory's seminal work, *Eye and Brain: The Psychology of Seeing* (Gregory 1966). Gregory suggested that our visual system relies on assumptions, such as the typical positioning of light sources above us, to resolve ambiguities in visual information. The light‐from‐above prior, a well‐documented perceptual bias, indicates that humans naturally assume light sources come from above, likely due to evolutionary exposure to sunlight as the primary illumination source (Gregory 1966; Ramachandran and Hirstein 1999). Research by Ramachandran (1988) and Adams et al. (2004) demonstrated that this prior is a robust and consistent bias, guiding the way we perceive forms and spatial relationships in visual scenes.

More recently, studies have examined the light‐from‐above bias in greater detail, revealing that this preference is not absolute but rather dependent on specific lighting angles. Research suggests that precise light directions influence attractiveness more effectively when positioned at certain optimal angles, rather than simply following a general top‐down illumination rule. For instance, Roelofs et al. (2022) found that a 45° superior (above) box light significantly enhances perceived attractiveness, as it emphasizes depth, highlights cheekbones, and reduces unflattering shadows. Conversely, a 90° overhead light is associated with reduced attractiveness, as it creates harsh shadows under the eyes and accentuates imperfections (Roelofs et al. 2022). These effects align with findings in other contexts such as portrait photography, theatre lighting, and cosmetic application, where controlling light angles enhances or diminishes aesthetic appeal. In a similar way, the study by Morgenstern et al. (2011) showed that individuals perceive faces as more aesthetically pleasing when they are lit from assumed natural angles, reinforcing the idea that perceptual expectations about light shape our aesthetic judgments.

### 2.1 Symmetry as Affordance

Symmetry is a fundamental concept people are familiar with, especially in two‐dimensional images and patterns. Reflective symmetry, for example, occurs when an object remains unchanged after mirroring. Human faces often exhibit vertical symmetry, and it is generally perceived as more attractive, possibly because it requires less cognitive effort for visual processing. However, a long‐standing debate in literature concerns whether symmetric faces are inherently more attractive than asymmetric ones. It is important to note that scientific literature often refers to symmetric faces as those resembling symmetrical configurations (artificial faces), as real human faces are never perfectly symmetrical (Borelli and Berneburg 2010). Indeed, numerous empirical studies showed that symmetric faces are typically rated as the most attractive (Baudouin and Tiberghien 2004). The reasons behind this preference may be influenced by psychological and cultural factors. From an evolutionary point of view, symmetrical faces are associated with genetic health and reproductive fitness (Borelli and Berneburg 2010; Penke et al. 2009). However, other studies suggest that facial symmetry is not always considered beautiful, and cultural factors may affect our perception of beauty (Little et al. 2011).

In addition, controversial findings challenge symmetry as a general rule for an attractive face, showing that some asymmetries, such as directional (Swaddle and Cuthill 1995) or peripheral asymmetry (Springer et al. 2007), can still be preferred. These findings resulted in being independent by job (medical doctors, art professionals, and people of other professionals were compared), age and wellbeing of participants (Springer et al. 2007).

While symmetry is often highlighted as a key determinant of beauty, its role is not entirely universally consistent across domains. Leder et al. (2019) interestingly found that symmetry is generally perceived as aesthetically pleasing, particularly in faces, where it is associated with health and genetic fitness. However, this preference was not universal across all domains, as participants rated mildly asymmetrical artistic compositions as more engaging, expressive, and visually interesting compared to perfectly symmetrical ones. Cultural differences also played a role, with Western participants showing a stronger preference for symmetry, especially in art and design, whereas East Asian participants displayed a greater tolerance for asymmetry, possibly reflecting artistic traditions such as Wabi‐Sabi, which values imperfection and irregularity. This suggests that expertise in art may manifest in different aesthetic preferences, potentially due to an increased appreciation for compositional complexity and deviations from predictable patterns. Moreover, these findings underscore the importance of collecting participant demographics in aesthetic studies, as expertise and education influence aesthetic judgments.

By contrast, the study by Springer et al. (2007), which examined the responses of medical doctors, art professionals, and general professionals to facial symmetry, yielded interesting results but did not explore how each group's background might have affected the findings. Including consistent information about participant populations (e.g., experts vs. non‐experts, or professional background) for all cited studies would greatly clarify how different factors—such as education, expertise, or professional training—may influence aesthetic judgments.

Makin et al. (2018) also examined cross‐cultural differences between English and Egyptian participants, integrating neuroimaging techniques. Their study assessed both behavioral preferences and neural responses to symmetrical stimuli. Using event‐related potentials, they measured sustained posterior negativity (SPN), a neural marker of symmetry processing, while participants viewed patterns with varying degrees of symmetry. The findings confirmed that symmetry is a strong predictor of aesthetic preference across both cultures, but Egyptian participants exhibited a greater appreciation for complex multi‐axis symmetry, likely influenced by cultural exposure to geometric patterns in Islamic art. Despite these variations, the SPN response remained consistent, suggesting a shared neural mechanism for symmetry perception.

Similar conclusions were drawn more recently in a comprehensive review on symmetry conducted by Bertamini and Rampone (2020). Specifically, here the authors pointed out how prolonged exposure to asymmetrical patterns may, over time, enhance an individual's appreciation for such forms, suggesting that aesthetic preferences are malleable and influenced by one's visual environment and education. The review delineated that familiarity and learned associations significantly impact aesthetic judgments.

This debate on symmetry extends across different disciplines. In the natural world, survival threats do not favor one side, so learning from a threat on one side benefits overall survival, indicating an evolutionary advantage for symmetry. This evolutionary significance may also account for why symmetry is not only appealing in nature landscapes, but also in abstract patterns and rhythms. For instance, Makin et al. (2012) used abstract dot‐patterns stimuli and found that symmetrical configurations were preferred over random configurations (for a review, see also Treder 2010). Despite its traditional association with beauty, studies show that, again, the point of view of the observer may be crucial in influencing a preference for symmetry.

Bertamini et al. (2019) also investigated the relation of symmetry vs. asymmetry debate, by comparing preferences for symmetrical and asymmetrical stimuli across various categories, including faces, geometrical objects, flowers and landscapes. Results showed that symmetry was generally preferred over asymmetrical stimuli, except for the case of landscape stimuli.

Symmetry also holds significant cultural and aesthetic value, permeating various forms of art. Its profound aesthetic significance is widely exploited in art, including visual arts, music, and rhetoric. In the pursuit of simplicity and power, symmetry is one of the most effective tools available. In testing artistic stimuli, Stieger and Swami (2015) found that participants consistently preferred symmetrical figurative stimuli (i.e., artistic photographs) over asymmetrical stimuli. This suggests that symmetry could be a crucial factor in how we perceive and judge the beauty of artworks.

Researchers suggested that preferences for symmetry relieve an aesthetic primitive, with symmetry being considered an innate, universal aspect of visual perception that elicits a positive response across different cultures and species (for a review, see Judd and Liu 2016).

Lucia et al. (2024) extended these investigations to abstract art by measuring visual scanning behaviors and aesthetic judgments of Rothko's paintings in different proportions. Results indicated that symmetric and quasi‐symmetric paintings were judged as more beautiful and more harmonious than those in other proportions.

However, despite many studies showed that symmetric stimuli are perceived as more beautiful, there is no counter proof that most of the stimuli judged as beautiful are symmetric. Many famous artworks universally acclaimed for their beauty are not symmetric (e.g., Baroque paintings, modern abstract compositions). Thus, symmetry can be considered as an aesthetic affordance: when present, it favors the fluidity and simplicity of visual processing, and it reduces cognitive workload. For instance, a symmetrical monument can be visually and even locomotory explored through symmetric movement patterns. Yet, the appeal of symmetry extends beyond its facilitation of visual processing. The human brain functions as a pattern seeker and recognizing a relationship between two parts of a stimulus can produce a sense of order and satisfaction (Bor 2012).

Overall, the relationship between aesthetic affordances and symmetry seems to be deeply embedded in both perceptual and ecological frameworks, underscoring the importance of symmetry in aesthetic experiences and environmental interactions, whereas its preference shall not be considered as absolute. It is important to consider how neural processing, cultural influences, and expertise shape aesthetic judgments. While symmetry serves as a powerful cue for beauty, it operates within a broader framework of perceptual and cognitive factors that contribute to aesthetic experience (Makin et al. 2012).

### 2.2 GR as Affordance

Another geometrical pattern often associated with beauty is the GR. The GR (i.e., phi, φ) is an irrational number approximately equal to 1.618, widely regarded as aesthetically pleasing. It occurs when a line is divided into two parts in such a way that the ratio of the whole line to the longer part is the same as the ratio of the longer part to the shorter part. This proportion has been used in art and architecture to create renowned visually compelling compositions, (e.g., Greek Parthenon's façade; Michelangelo's *Creation of Adam*).

The GR shares analogous properties to symmetry: both are geometric characteristics widely present in nature (Iosa et al. 2018), including physiological rhythms such as that of human gait (Iosa et al. 2013) and heart beating (Yetkin et al. 2013). Both proportions may facilitate visual processing to the extent that they may function as ecological affordances (De Bartolo et al. 2022). This suggests a deep connection between these geometric proportions and the perception of beauty, as artists have intuitively recognized since ancient times.

For instance, De Bartolo et al. (2022) investigated aesthetic preferences by presenting participants with images of humans, sculptures, paintings, digital humanoids and geometrical shapes, in different proportions (1.5, GR, 1.8). Results showed an explicit preference for the GR in human images, sculptures, and paintings. Notably, measures of eye movements revealed that shorter dwell times correlated with greater aesthetic preference. Similarly to symmetry, GR may facilitate visual processing by establishing a precise proportion between the whole stimulus and its wider part, which in turn maintains the same ratio with the smaller part. Due to this ease of processing, it has been suggested that GR could also be considered an ecological affordance (De Bartolo et al. 2022).

Salera et al. (2024) built upon these findings by investigating the implicit nature of GR preferences. They reproduced the experimental setup used by Makin et al. (2012) that tested aesthetic preferences for symmetry, substituting symmetric dot patterns with GR‐based dot patterns and comparing them to analogous stimuli in random proportions. Using an implicit association task, participants showed a facilitation when GR stimuli were implicitly associated with positive words, compared to when words had a negative valence.

In contrast, Stieger and Swami (2015) found that symmetry elicited a greater impact on participants' aesthetic judgments compared to GR, suggesting a higher and simpler level of harmony for symmetry. Similarly, Lucia et al. (2024) suggested that symmetry might be the preferred form of regularity, potentially leading to an after effect. In fact, exposure to highly regular stimuli, such as symmetric patterns, could make fewer regular patterns even more irregular, reducing their perception of regularity (Ouhnana et al. 2013), despite GR being harmonic. The same study also revealed that symmetric and quasi‐symmetric stimuli were rated as more harmonious than those conforming to the GR or exceeding it. However, these explicit ratings were not significantly correlated with visual scan behavior.

## 3 Internalization of Affordances

In 1984, Roger Shepard defined invariants as the stable properties or features in mental representations that remain constant even when these characteristics that are roughly persistent in the world disappear or undergo transformations such as rotation or scaling (Shepard 1984). These invariants play a crucial role in visual perception, providing a consistent framework that allows us to accurately recognize and interpret objects and their spatial relationships, even when their appearance changes from different angles or perspectives. In other words, invariants help us maintain a coherent understanding of the world, preserving certain key perceptual features in our mental imagery despite external variations (Shepard 1984).

The use of invariants is important for aiding visual perception in ambiguous conditions. In fact, to overcome ambiguities, the visual system uses prior knowledge of ecological invariants. For instance, the assumption that light comes from above can help us determine whether a shape is convex or concave (Mamassian and Goutcher 2001).

Another invariant could be the disposition of specific characteristics to identify an object. For example, human faces are characterized by having two eyes placed symmetrically with respect to an axis, with the nose above the mouth. When a face is presented upside–down, this inversion makes it difficult to evaluate its attractiveness accurately. A typical example is the Thatcher effect, which occurs when a grotesque face (digitally modified) is shown upside down, impairing face perception and making the face appear normal (de Haas and Schwarzkopf 2018).

Moreover, evidence shows that the intensity of this effect depends on the face orientation angle (Stürzel and Spillmann 2000). Orientation is not only related to kinematic aspects, but also to kinetic ones, because the orientation of an object is often defined with respect to gravitational vertical axis. Gravity is one of the most well‐known invariants that was reported as internalized, since people expect that heavier objects will require greater applied force to be lifted up, and that objects fall down along vertical direction accelerating (at constant rate of 9.81 m/s^2^) (Zago et al. 2004).

### 3.1 Internalization of Symmetry and GR

The distinction between an affordance and an internalized invariant lies in their recognition and expectations. An affordance is perceived when encountered in the surrounding environment, while an invariant is expected even if not present in a stimulus.

Despite our visual system being very sensitive to symmetry, the observation that quasi‐symmetrical stimuli are often treated as symmetric ones (Lucia et al. 2024), may suggest that symmetry is an internalized feature, recognized even when not present.

One of the most cited aspects in affordance theory is the “graspability” of an object, which refers to how object design facilitates its use. If an object is symmetrical, its center of mass is along the symmetrical plane, facilitating the understanding of where the force should be applied to minimize energy consumption, annulling the torques. Bingham and Muchisky (1995) suggested that symmetry may facilitate the identification of the center of mass of an object and hence its graspability.

The human body is probably symmetrical, likely because the two sides (left and right) developed under the same gravitational field. Conversely, along the cranio‐caudal axis, the body developed in proportions conform to the GR with the navel (the point from which the fetus is alimented) coinciding with the center of mass of the body, and dividing the upper and lower body in the same proportion as the lower and the whole body (a characteristic possible only dividing the stature in golden proportion) (Iosa et al. 2018).

Hence, humans may have internalized these geometrical invariants because they are familiar with them, are helpful in interpreting and predicting reality, and are associated with specific regularities.

Lucia et al. (2024) tested the effect of orientation on abstract artworks of Rothko to investigate how symmetry and the GR in abstract art influence judgments of pleasantness and harmony. Perceived harmony, but not the pleasantness of the stimuli, resulted associated to its orientation, with the left–right symmetry judged as more harmonious. Eye dwell time (a variable often associated with attention) varied based on the stimulus orientation, and observers spent more time looking at the above area compared to the below area. This finding can be interpreted according to the concept of “graspability” associated to affordances: subject may find it easier to interact with objects when they are positioned above others rather than below (Zago et al. 2004). Interestingly, for horizontally symmetric stimuli (i.e., with a vertical axis of symmetry), the dwell time was equally distributed in the two areas (50%–50%) (Lucia et al. 2024). But when symmetry was vertical (with a horizontal axis of symmetry), subjects spent longer time looking at the above area, in a golden proportion to the below area. Conversely, when the stimulus conformed to the GR, the dwell time was symmetrically distributed between the small above area and the larger area below. Finally, when the stimulus was rotated, this pattern is lost, and the dwell time is proportional to the covered area. This study provides novel insights into the interplay between visual regularity and aesthetic experience in abstract art (Adams et al. 2004). Furthermore, by examining visual scan behavior, the study highlights potential links between high‐level aesthetic experience and low‐level visual attention processes.

The idea that some stimuli can be visually scanned using the golden proportion (even if the stimulus is not in GR), had been already suggested (Brincker 2015). Symmetry and GR could be considered as two affordances (Tsikandilakis et al. 2019; Arias 2019) which, according to the theory of internalization of ecological invariants (Gallagher 2011), mean that individuals could scan according to these proportions in search of regularities. Most of the symmetric stimuli we encounter exhibit horizontal left–right reflections (such as human and animal bodies, furniture, cars), whereas GR proportion is mainly on the vertical direction.

## 4 Toward a Kinetic Approach to Beauty Perception

A possible interpretation linking aesthetic preferences to specific orientations of stimuli may relate to humans' internal model of gravity. This cognitive framework is developed through our lifelong interactions with the physical world, allowing us to anticipate movements such as falling, rolling, and other gravitational effects (Duhamel et al. 1992; Jeannerod 1997; Wolpert et al. 1998). By leveraging this model, our brain predicts how objects should behave in a gravitational field, making the processing of visual information smoother and more efficient. This phenomenon, known as “processing fluency,” refers to the ease which sensory information is processed by our cognitive system. High processing fluency occurs when stimuli aligning with our expectations, derived from the internal model of gravity, leads to a more enjoyable and aesthetically pleasing experience. For example, a painting or animation that accurately depicts gravitational effects will likely be perceived as more appealing, as it conforms to our ingrained expectations, requiring less cognitive effort to process.

The novelty of our approach points out that symmetry can be aesthetically preferred not only because we are familiar with it and because it facilitates visual processing, but also because it facilitates the possible interaction with the object, such as graspability, for which is important to correctly predict the position of the center of mass. The same may occur for the golden proportion along the vertical axis. Thus, from a previous interpretation focused on perception of static and kinematic features, we suggest the need to consider actions and kinetics. Beauty can be associated with the concept of balance that should also take into account forces. A beautiful symmetric face can lead not only to its symmetric visual scan, but also to symmetric movements for caressing it.

In neuroaesthetic fMRI studies, researchers have reported intriguing insights into how the perception of art can activate brain regions associated with embodied cognition, thus revealing unconscious cognitive processes. For instance, when examining Michelangelo's Prisoners sculptures—depicting individuals in the tense act of emerging from rock—viewers showed activation in brain areas related not only to sensory functions, but also to motor ones, such as the premotor and motor cortices. This suggests that participants engage in an embodied response that unconsciously reflects the physical effort and tension depicted in the sculptures. These findings indicate that viewers not only experience a profound emotional connection with renowned art but also develop deep empathy (Di Dio et al. 2011).

The importance of kinetic aspects in beauty perception is highlighted by the fact that the vision of an art masterpiece activates sensorimotor networks, specifically the mirror neuron networks. These networks can be activated by the following: the actions performed by painted persons (Freedberg and Gallese 2007); the ideal possibility to move in the scene or using the depicted objects (Wolpert et al. 1998); and even to the empathetic engagement activating simulation of the motor program that corresponds to the gesture implied by the trace done by the painter into the observer (Knoblich et al. 2002; Freedberg and Gallese 2007).

Similar evidence is observed with non‐figurative art. In a study investigating abstract art, Umiltà and colleagues (2012) found that participants viewing Lucio Fontana's slashed canvases engaged neural circuits involved in embodied cognition and body schema. This suggests that viewers mentally simulate the act of slashing or interacting with the perceived tension in the artwork. Overall, these findings support the complex interplay between viewers and artwork, suggesting that much of this engagement occurs beyond conscious awareness, and take into account motor actions and the required dynamic effort, all aspects related not only to the kinematic features of the stimuli, but also to the kinetic ones.

### 4.1 The Role of Other Invariants on Beauty Perception

This perspective review suggests that environmental invariants and affordances may affect the general model of beauty perception. We focused on symmetry and the GR, two widely studied proportions in aesthetic perception, as emblematic features of broader kinetic principles of ecological perception.

For years, visual perception was primarily thought to be based only on static or kinematic features of objects and scenes, but with the discovery of the model of gravity this approach changed, and kinetic invariant started to be embedded in visuo‐motor control (Zago et al. 2004; McIntyre et al. 2001). Analogously, it is possible that symmetry and GR are not only preferred for their geometric harmony, but also because they function as kinetic internalized affordances.

Other environmental invariants that are internalized could influence our perceptions, for example, the common direction of light from above (Mamassian and Goutcher 2001). A technique used in horror movies is to light faces from below to alter the common perception of the observer. Our perception of beauty and our aesthetic judgment are influenced by lighting direction because the typical positioning of light sources are above us, and we have internalized this information to resolve ambiguities in visual processing (Gregory 1966; Ramachandran and Hirstein 1999). More recently, studies have examined the light‐from‐above bias in greater detail, revealing that this preference is not absolute but rather dependent on specific optimal lighting natural angles (Morgenstern et al. 2011; Roelofs et al. 2022).

These environmental invariants are not completely independent, and their internalizations could be intertwined in our perception of the external world. Recent advancements have further refined our understanding of how gravitational cues interact with the light‐from‐above prior, revealing that our perception of light direction is not only based on visual assumptions but also on bodily orientation relative to gravity (Barnett‐Cowan et al. 2018). Ultimately, the preference for light from above in beauty reflects deeper cognitive and perceptual mechanisms, shaped by both evolutionary predispositions and environmental adaptations.

The kinetic approach proposed in this review could also be influenced by the fact that stimuli that are in accordance with ecological invariants are more common and subjects could have a greater experience of them. This topic is related to the difference between affordances (that is present in the interaction between perceived object and perceiving subject) and internalized invariants (present in the subject even when not present in the object). The distinction is difficult because the most frequent typology of the stimulus is related to its kinetic properties. Further research could design more specific studies for testing the kinetic approach to beauty perception proposed in our study, for example, testing if subjects would prefer an equilateral triangle when the lower part is on one of its sides or when it is one of its vertices. The former would be in line with a kinetic approach of beauty perception because if this triangle is an object in a gravitational field, it is stable, whereas the latter is a condition not in line with the proposed approach. For now, the results of Lucia et al. (2024) about the effect of orientation on abstract paintings of Rothko seemed to suggest a kinetic interpretation of aesthetic judgments.

This perspective review introduces a new kinetic approach as a possible explanation for aesthetic preferences toward stimuli that are symmetric or follow the GR. This does not imply that beauty perception is solely related to kinetic invariants, as previously demonstrated for the direction of light that may influence the aesthetic judgment because of a familiarity bias.

The proposed theory suggests incorporating kinetic aspects into the study of beauty perception, expanding visual processing to include visuo‐motor control, even in the absence of actual movement execution. This aligns with the “4E” cognition theory (Carney 2020), which posits that cognition is Embodied, Embedded, Extended, and Enacted, closely linked to potential interactions with the surrounding environment. Just as visual perception should consider not only the geometrical and kinematic properties of objects (and our body) but also their kinetic characteristics, the perception of beauty could follow the same process. For instance, another environmental feature proposed as a possible invariant is friction (Hubbard 1995). Aesthetic preferences for smoothed skin (Tsankova and Kappas 2016) could, on one hand, be associated with an implicit link between skin smoothness and health. On the other hand, they could be related to a reduction of friction during interaction, such as during caressing. Further studies designed with specific experiments are needed to test our theory, particularly for these less explored invariants.

## 5 Conclusion

In the first part of this perspective review, we addressed some open questions about beauty perception. Thus, we proposed a theoretical framework that could help to clarify some of the incongruent findings reported in literature.

We interpreted existing data from the literature on the visual perception of stimuli characterized by symmetric patterns or golden proportions between their parts, aesthetic judgments of these stimuli, and their possible internalization as invariants within an environment governed by gravity and kinetic laws. The proposed theoretical framework appears to be consistent with the data reported in scientific literature and, furthermore, seems to resolve some experimental discrepancies while shedding light on certain unclear aspects of beauty perception and aesthetic judgments.

First, we questioned whether beauty is an objective quality or a subjective feature, which depends on the observer. By interpreting beauty as an affordance, we overcome this dichotomy, as an affordance relates neither to a set of characteristics of the stimuli nor a subjective interpretation of them, but rather a relationship between the object and the subject.

Next, we explored whether beauty is an aware cognitive process, an unaware perception or an emotion. An affordance may act across different levels. For example, Salera et al. (2024) showed that GR is implicitly associated with positive words, while De Bartolo et al. (2022) found an explicit preference for the GR, but only for some specific kinds of stimuli related to anthropometric dimensions. Furthermore, Vara Sánchez (2022) suggested that affordances are not merely about how we interact with objects, but also about how they influence our emotional states. This reinforces the idea that interaction between the individual and the object extends beyond potential actions and includes potential affective responses.

Moreover, the ancient Greek introduced the concept of *kalokagathia*, the identity of beauty and goodness. While this may contribute to psychological bias, it may also be related to the internalization of symmetry and GR as ecological invariants related to the concept of balance and justice (Suleiman 2017). In the Ultimatum Game task, participants are asked to choose how much of a total hypothetical amount of money they are willing to share with another imaginary participant that could refuse the offer if judged unfair, leading to a loss of money for both. Hence, they should opt for a fair division of money to reduce the risk of the other participant's refusal. It was reported that the choices judged as the fairest were the symmetric divisions (50%–50%) (Yetkin et al. 2013) and the division in golden proportion (61.8%–38.2%), as the proportion renounced by the participant to the amount gained is the same as the proportion between the total amount and the one retained (Schuster 2017). The use of symmetry and golden proportion in contexts different from vision and geometry (i.e., the Ultimatum game, which refers to the theory of games and economic behaviors) may suggest their internalization, justifying their use in different contexts.

Because the presence of horizontal symmetry and vertical golden proportion in anthropometric dimensions and other characteristics of the surrounding environment can be caused by force balance, we provided a kinetic interpretation of this internalization. In fact, symmetry is often considered an intrinsic property of an object that may facilitate the visual processing of the observer. However, this binary relationship does not take into account an important factor: the surrounding environment and its physical laws. Animal bodies and plants have evolutionarily developed a shape that is symmetric with respect to the vertical axis because the gravitational field defines verticality. Conversely, along the vertical direction, some organic structures (such as the human body) exhibit the GR, with a shorter part above the center of mass and a longer one below it, in the same proportion as the long part to the whole length. This allows for better mass balance along the vertical direction with respect to the gravitational field. Even the motor control of inertial parameters can be simplified due to this harmonic arrangement of body masses.

This relationship with balance could also explain why symmetry and the GR are not only associated with aesthetic preferences but also with ethical preferences (Schuster 2017). This approach is particularly innovative because it suggests taking into account kinetic features in visual processing, rather than limiting their analyses only the static or kinematic characteristics of scenes and objects. Zago et al. (2004) showed that expected dynamics (i.e., kinetics) overrides measured kinematics in the visual processing of object movements, and it is possible that the same applies to visual processing in aesthetic judgments.

It should be pointed out that this perspective reviews how symmetry and GR influence aesthetic preferences but does not clarify all the aspects about the definition of beauty, a complex construct that also limits the diffusion of valid scales and tools allowing its assessment. As Gibson and Shepard emphasized, humans are not merely observers but active explorers of their surroundings. Our symmetric hands may prefer to caress symmetrically smooth faces, and objects with golden proportions along vertical axes might be perceived as more balanced and, consequently, easier to handle.

This review introduces a new theoretical framework that may help clarify some of the long‐standing unsolved questions about perception of beauty. However, despite being developed based on published experimental data, the proposed kinetic approach to beauty perception should be tested in further studies specifically designed to validate this theory.

## Author Contributions

MI: conceptualization and revision; MPL: literature revision and first draft writing; CS: first draft writing and revision. All authors have read and agreed to the published version of the manuscript.

## Conflicts of Interest

The authors declare no conflicts of interest.

### Peer Review

The peer review history for this article is available at https://www.webofscience.com/api/gateway/wos/peer‐review/10.1111/ejn.70119.

## Disclaimer/Publisher's Note

The statements, opinions and data contained in all publications are solely those of the individual author(s) and contributor(s) and not of MDPI and/or the editor(s). MDPI and/or the editor(s) disclaim responsibility for any injury to people or property resulting from any ideas, methods, instructions or products referred to in the content.

## Data Availability

No original data are reported in this review.
